# Rift Valley fever virus vaccination induces long-lived, antigen-specific human T cell responses

**DOI:** 10.1038/s41541-020-0166-9

**Published:** 2020-02-28

**Authors:** Jessica R. Harmon, Dominique J. Barbeau, Stuart T. Nichol, Christina F. Spiropoulou, Anita K. McElroy

**Affiliations:** 1 US Centers for Disease Control and Prevention, Viral Special Pathogens Branch, 1600 Clifton Rd, Atlanta, GA 30333 United States; 2 University of Pittsburgh, Division of Pediatric Infectious Disease, 3501 Fifth Ave, Pittsburgh, PA 15261 United States

**Keywords:** Cellular immunity, Vaccines, Virology

## Abstract

Rift Valley fever virus (RVFV) is a zoonotic arbovirus of clinical significance in both livestock and humans. A formalin-inactivated virus preparation was initially developed for human use and tested in laboratory workers in the 1960s. Vaccination resulted in generation of neutralizing antibody titers in most recipients, but neutralization titers waned over time, necessitating frequent booster doses. In this study, T cell-based immune responses to the formalin-inactivated vaccine were examined in a cohort of seven individuals who received between 1 and 6 doses of the vaccine. RVFV-specific T cell responses were detectable up to 24 years post vaccination. Peripheral blood mononuclear cells from this cohort of individuals were used to map out the viral epitopes targeted by T cells in humans. These data provide tools for assessing human RVFV-specific T cell responses and are thus a valuable resource for future human RVFV vaccine efforts.

## Introduction

Rift Valley fever virus (RVFV) is a mosquito-borne hemorrhagic fever virus that causes morbidity and mortality in humans and livestock. It was first identified in 1931 in Kenya after isolation from a sheep in the Rift Valley,^1^ and has caused disease throughout continental Africa, Madagascar, Yemen, and Saudi Arabia.^2^ Reports of vector capacity in North America in multiple genera of mosquitoes have suggested that RVFV could be a potential threat to the US;^3^ given the rapid global spread of Zika virus in 2015, transmitted by only one mosquito genus, the risk of global spread of RVFV is not insignificant.

RVFV is largely a veterinary pathogen that infects sheep, goats, and cattle. Mortality of up to 90% has been reported in newborn animals and up to 30% in adult animals.^4^ Consistent with its high pathogenicity in juvenile animals, RVFV is also abortigenic: 40–100% of animal pregnancies will end in spontaneous abortions during an RVFV outbreak, leading to “abortion storms”.^1,4^ Furthermore, livestock caretakers, herders, and veterinarians are exposed to RVFV in the process of caring for affected animals and handling infected tissues, since both blood and amniotic fluid contain high quantities of virus.

In addition to transmission through contact with infected livestock, RVFV can be transmitted to humans by the bite of an infected mosquito. Infected individuals typically develop a mild disease consisting of fever, malaise, and myalgia. A small percentage of individuals, however, develop severe disease that manifests as hepatitis, encephalitis, retinitis, or hemorrhagic fever, which are the hallmarks of fulminant RVFV clinical disease. The overall case fatality rate is estimated at less than 3%, but has been as high as 50% in some outbreak settings (https://www.who.int/news-room/fact-sheets/detail/rift-valley-fever).

A formalin-inactivated vaccine preparation was originally generated in the 1960s and tested in humans for safety and for generation of a neutralizing antibody response (considered to be the correlate of protection).^5,6^ Since then, other formalin-inactivated vaccine preparations have been made and tested in humans with similar results, but the vaccine is no longer being administered and only limited quantities exist.^7–11^ Studies of the immune response to vaccination with these preparations have been limited to assays of neutralizing antibody responses. Recent data obtained in the mouse model suggested that the T cell-mediated immune response, not just the antibody response, could be important in protecting animals (and by extension, humans) from disease.^12,13^ Additionally, two reports assessed HIV-positive patients who acquired RVFV infection; these patients exhibited increased frequency of central nervous system (CNS) symptoms and a significantly higher case fatality rate than HIV-negative patients,^14,15^ suggesting the importance of CD4 T cells in preventing CNS manifestations and decreasing the severity of RVFV disease. Given these findings, it was of interest to quantitate and characterize T cell-mediated immune responses in humans. The limited availability of RVFV human acute or convalescent peripheral blood mononuclear cell (PBMC) specimens made recruiting RVFV-vaccinated individuals a more feasible route for initial studies to establish methods. Additionally, a recent move by the Coalition for Epidemic Preparedness Innovations (CEPI) to develop a RVFV vaccine for human use made the need for assaying human T cell responses against RVFV antigens even more urgent (https://cepi.net/research_dev/priority-diseases/). Therefore, we quantitated and characterized T cell-mediated memory responses that were generated in humans after vaccination with the formalin-inactivated RVFV vaccine and compared these results with those obtained by serologic methods.

## Results

Seven individuals who received the RVFV formalin-inactivated vaccine were enrolled in this study. They ranged in age from 37 to 61 years; four were male and three were female. They received between 1 and 6 doses of the vaccine 7–24 years prior to sample collection for this study (Table 1).

**Table 1:** Details of vaccine recipients.

Vaccinee	RHI1	RHI2	RHI3	RHI4	RHI5	RHI6	RHI7
Years since last vaccine	14	7	24	7	7	16	14
Total doses of vaccine	4	3	6	6	4	3	1
Genetic background	Caucasian American	Caucasian American	Caucasian European	Caucasian American	African American	Caucasian American	Caucasian American
Age	55	37	61	48	46	43	49
Sex	F	M	M	M	M	F	F
HLA-A2+	Yes	Yes	No	Yes	Yes	Yes	No

### Serologic studies

**Fig. 1:**
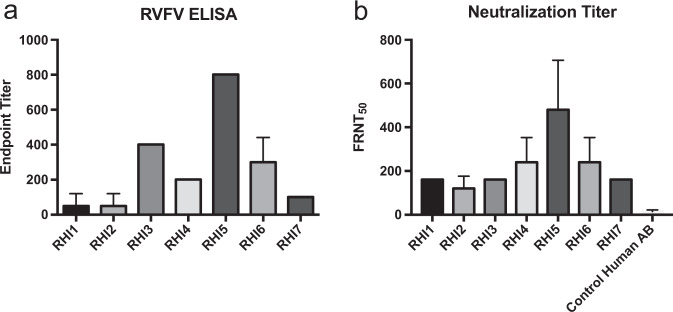
RVFV humoral responses in vaccine recipients. **a** ELISA assays and **b** neutralization assays were performed on serum samples from all vaccine recipients. The ELISA endpoint titer is the dilution of serum at which the signal was three standard deviations above that of the average of the negative controls. The mean and standard deviation of technical replicates is shown. The focus reduction neutralization titer 50 (FRNT_50_) is the dilution of the serum that neutralized 50% of the input foci. A human serum control is also shown for the neutralization assay since low-level, non-specific neutralization can be seen in this sample.

Earlier studies of the human immune response to this same formalin-inactivated vaccine only measured humoral responses, as detected by virus neutralization assays.^5,6,11^ Here, we performed both ELISAs and neutralization assays to evaluate the humoral response in vaccine recipients. All vaccine recipients had detectable ELISA responses against a total RVFV lysate (Fig. 1a), but the magnitude of those responses varied considerably. This assay utilized whole cell lysate from RVFV-infected cells, which contain all viral antigens and should detect any virus-specific antibodies.

A 50% focus reduction neutralization assay (FRNT_50_) was used to assess the neutralizing activity in vaccine recipient serum samples (Fig. 1b). Similar to the data obtained from ELISA assays, FRNT_50_ activity varied significantly. Published studies related to this vaccine reported that neutralizing activity waned rapidly over time: 1 year post vaccination, only 30% of the recipients had antibody titers that were considered protective, which the investigators defined as greater than 1:40 by 80% plaque reduction neutralization test (PRNT_80_).^11^ When we analyzed the FRNT data for 80% neutralization, only RHI5 and RHI6 had titers of 1:40, the rest of the vaccine recipients had FRNT_80_ titers less than 1:40. Therefore we presented the data as FRNT_50_ so that the breadth of responses among the donors could be appreciated. We observed no statistically significant correlation between ELISA or FRNT_50_ titers and number of vaccine doses received or number of years since last dose (Spearman r of 0.3890 for doses and Spearman r 0.1983 for years).

### Experimental T cell epitope mapping

A series of overlapping peptides was generated from the entire open reading frames of the three viral structural proteins: nucleocapsid (N) and glycoprotein (cleaved post translationally into Gn and Gc). Peptides were 15-mers with 11-mer overlaps as per the protocol of Hoffmeister et al.,^16^ resulting in 59N peptides and 264 GP peptides (140 from Gn and 124 from Gc). Using a 2-dimensional map, 16 peptide pools were generated for N, 24 for Gn, and 23 for Gc. PBMCs from vaccine recipients and negative control donors were incubated with peptide pools and evaluated for cytokine production using intracellular cytokine staining (ICS) in a flow cytometry assay. Many positive peptides were identified using these assays, but the low frequency of events indicated that RVFV-specific T cells were very rare in these vaccine recipients. Therefore, in order to increase the sensitivity of the screen, the peptide pools were also screened against PBMCs from vaccine recipients and negative control donors using an IFN-γ ELISPOT assay. Positive pools from either assay were compared to the 2-dimensional grids for each antigen’s peptide pools to deconvolute the data, and a listing of all positive peptides for each donor was generated. Down-selecting individual peptides based upon the ICS and ELISPOT peptide pool mapping assays resulted in 44 peptides for N, 92 peptides for Gn, and 83 peptides for Gc. Any positive peptide from either of these screens was then evaluated in an individual peptide assay.

**Fig. 2:**
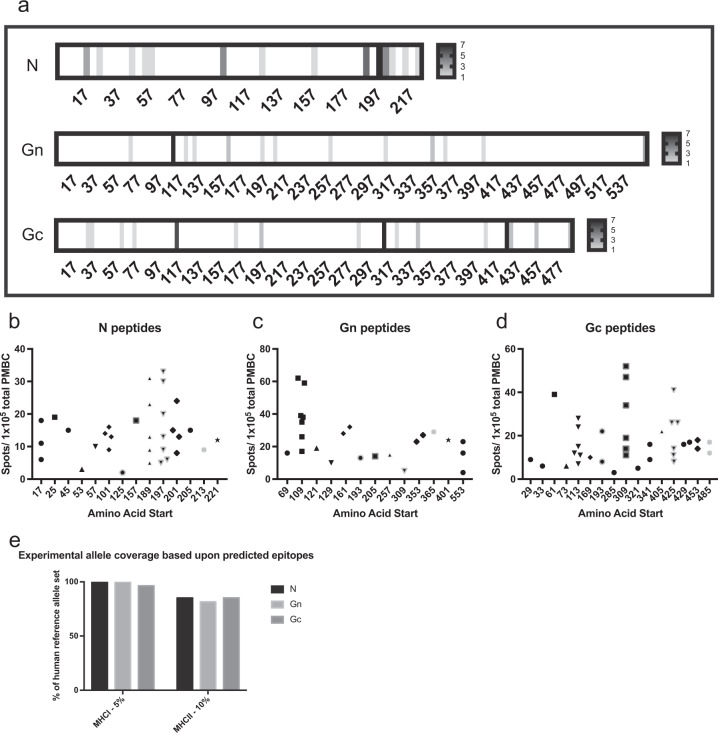
Mapping RVFV-specific T cell epitopes in vaccine recipients. **a** A graphical depiction of each viral structural protein (N, Gn, and Gc), with a heatmap to indicate the number of donors whose PBMCs recognized the epitope based upon individual peptide assays performed by ELISPOT. The magnitude and amino acid location for all positive donors for the three structural proteins: **b** N, **c** Gn, and **d** Gc. **e** Experimentally determined epitopes correlate well with bioinformatic prediction of epitopes recognized by the common human allele reference set.

IFN-γ ELISPOT assays were then repeated for all vaccine recipients and control PBMCs using each individual peptide. From these data, a list of peptides that were positive in any of the seven donors from each protein was compiled. This resulted in 14 peptides for N, 13 peptides for Gn, and 16 peptides for Gc (Table 2). The final Gn peptide contains the GnGc cleavage site (noted by underlined text). In all three proteins, some epitopes were recognized by all seven donors in IFN-γ ELISPOT assays (Fig. 2a). The number of spots produced per 1 × 10^5^ PBMCs from each donor in these targeted epitopes varied (Fig. 2b–d).

**Table 2:** Down-selected empirically determined RVFV epitopes.

N (aa start #)	Gn (aa start #)	Gc (aa start #)
DRNEIEQWVREFAYQ (17)	KFPLFQSYAHHRTLL (69)	ALIRAGSVGAEACLM (29)
VREFAYQGFDARRVI (25)	CMKEKLVMKTHCPND (109)	AGSVGAEACLMLKGV (33)
YGGADWEKDAKKMIV (45)	PNDYQSAHYLNNDGK (121)	SSELSCREGQSYWTG (61)
DAKKMIVLALTRGNK (53)	YLNNDGKMASVKCPP (129)	WTGSFSPKCLSSRRC (73)
MIVLALTRGNKPRRM (57)	SLKKGSYPLQDLFCQ (161)	VGESTTMRENKCFEQ (113)
ELTLSRVAAALAGWT (101)	EVGVQALKKCDGQLS (193)	LTLEITDFDGSVSTI (169)
WLPVTGTTMDGLSPA (125)	QLSTAHEVVPFAVFK (205)	TNWGSVSLSLDAEGI (193)
LPGDYLRAILDAHSL (157)	SGQTKRELKSFDISQ (257)	VFERGSLPQTRNDKT (285)
KEEVAATFTQPMNAA (189)	GSGIVQIQVSGVWKK (309)	VQAFSKGSVQADLTL (309)
TQPMNAAVNSNFISH (197)	ITKCEPHGLVVRSTG (353)	LTLMFDNFEVDFVGA (321)
NAAVNSNFISHEKRR (201)	STGFKISSAVACASG (365)	AAFLNLTGCYSCNAG (341)
NSNFISHEKRREFLK (205)	QSSGGDIGVHMAHDD (401)	EEFMYSCDGDERPLL (405)
KRREFLKAFGLVDSN (213)	LIVSYASACSELIQA (553)	IAIDPFDDRREAGGE (425)
FGLVDSNGKPSAAVM (221)		PFDDRREAGGESTVV (429)
		FDWFSGLMSWFGGPL (453)
		FLLIYLGRTGLSKMW (485)

### Predictive T cell epitope mapping

The T cell epitope prediction tool from the Immune Epitope Database and Analysis resource (www.IEDB.org)^17^ was used to determine which RVFV epitopes would be bound by the human leukocyte antigen (HLA) reference set of MHC class I and II molecules. The HLA allele reference sets are a predefined set of 27 MHCI and MHCII alleles that represent >97% (MHCI) and 99% (MHCII) of the human population.^18,19^ Each RVFV protein sequence (N, Gn, and Gc) was input into the tool; the IEDB-recommended 2.19 prediction method was selected along with the HLA allele reference set for each class. The output from each analysis (6 in total) was exported to Excel and the top 5% (MHCI) or 10% (MHCII) of predicted epitopes for each protein were examined for the epitope sequences defined by the experimental dataset (peptides in Table 2). A more stringent cutoff was chosen for MHCI since the algorithms for MHCI are much more robust than those for MHCII. The number of reference set alleles that were represented in the experimental dataset was identified for each protein for each class. Overall, the panel of peptides identified by the experimental dataset represented >80% of the allele reference set for MHCII and >90% of the allele reference set for MHCI (Fig. 2e), suggesting that this panel of experimentally determined RVFV-specific peptides based upon a small cohort of individuals would likely be applicable to the larger human population and useful for measuring RVFV-specific T cell activity in human vaccine studies.

### Activation, proliferation, and functionality of virus-specific T cells

**Fig. 3:**
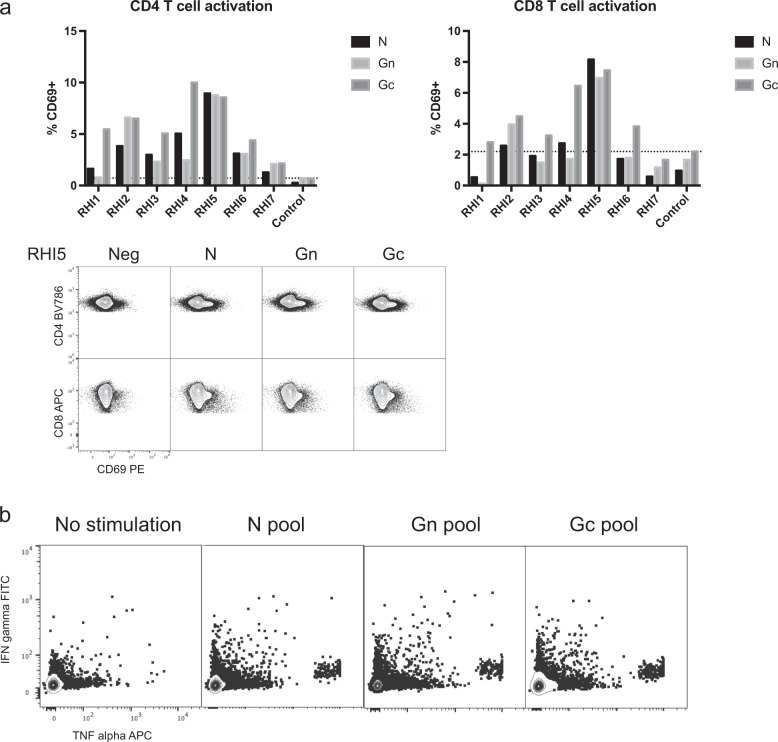
Functional assays of RVFV-specific T cells in vaccine recipients. **a** In vitro exposure of PBMCs from most vaccine recipients to the down-selected panel of peptides (Table 2) for each structural protein resulted in upregulation of the T cell activation marker CD69 on both CD4 and CD8 T cells. The dotted line shows the background frequency based upon the highest frequency of expression observed in the negative control. Representative raw flow plots depicting CD69 expression are shown for the donor with the highest RVFV-specific T cell activity (RHI5). **b** PBMCs from RHI5 also expressed IFN-γ and TNF-α following peptide stimulation.

Using these down-selected pools of peptides for N, Gn, and Gc, we evaluated the functionality of RVFV-specific T cells in vaccine recipients. Total PBMCs from each vaccine recipient and a negative control donor were incubated separately with each viral protein-specific pool or left unstimulated. Three days later, cell activation was assessed by staining for CD69. After peptide stimulation, most vaccine recipient donors had CD69+CD4 and CD8 T cell frequencies above the background level seen in the negative control donor (Fig. 3a, b). Overall, the frequency of CD69 staining was greater in CD4 T cells than in CD8 T cells, and donor RHI5 had the most robust responses of all donors. Flow cytometry plots of CD69 staining on both CD4 and CD8 T cells are shown for RHI5. Assays to evaluate proliferation following peptide stimulation were also performed using both CellTrace labelling and Ki-67 staining, but no T cell proliferation was detected following RVFV peptide stimulation.

We also assessed the functionality of RVFV-specific T cells by intracellular cytokine staining following in vitro stimulation with the same down-selected peptide pools. Three negative control donors were included for comparison. As a group, the vaccine recipients did not have a statistically significant increase in cytokine expression following peptide stimulation compared to controls. However, RHI5 was again an outlier in this cohort and exhibited expression of TNF-α and IFN-γ by CD8 T cells upon stimulation with RVFV peptide pools (Fig. 3b).

### Assessing virus-specific cells by pentamer staining

**Fig. 4:**
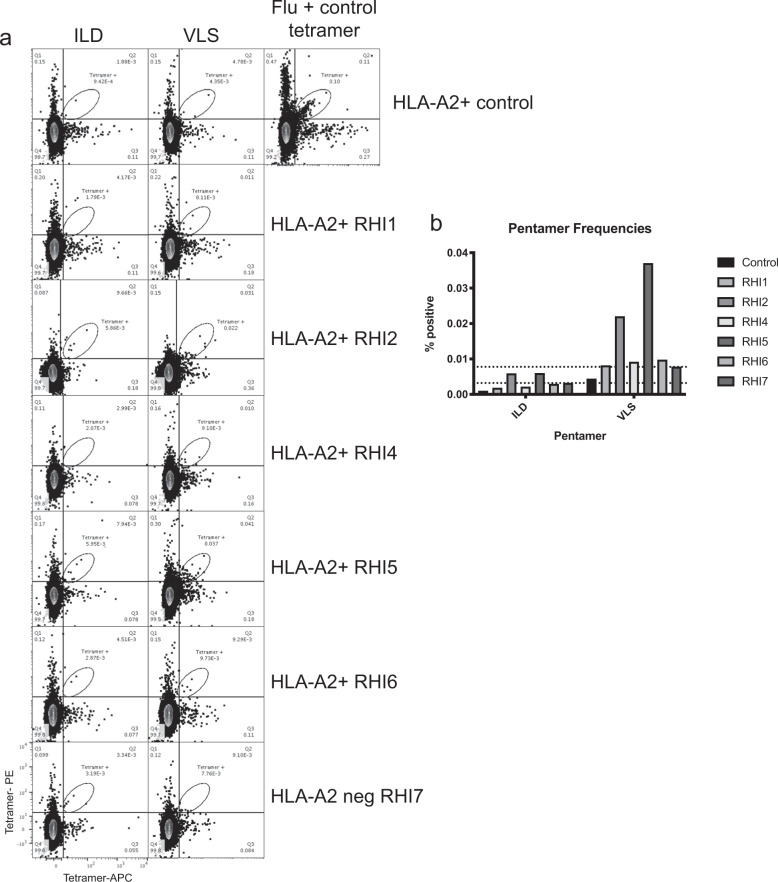
Pentamer staining on HLA-A2-positive donors. **a** Pentamers against 2 RVFV HLA-A2-directed epitopes (ILD and VLS) were labeled on both APC and PE and were incubated with PBMCs from various donors. A sample from a control donor who is HLA-A2-positive but unvaccinated is also included; this donor has influenza-specific CD8 T cells but no RVFV-specific CD8 T cells. The pentamer frequencies for each donor are shown in **b**. Dotted lines show the background value for each pentamer based upon the background signal observed in the HLA-A2-negative donor, RHI-7.

Permission for genetic testing was not obtained during the consent process so it was not possible to determine each donor’s HLA type via genetic testing. However, given that HLA-A2 is a common MHCI allele, donors were evaluated for HLA-A2 expression by flow cytometry, and 5 of the 7 were HLA-A2 positive (Table 1). Two HLA-A2-directed RVFV N immune dominant epitopes were previously defined by Xu et al., who used N-transduced dendritic cells to prime CD8 T cells from HLA-A2 donors.^20^ The 2 immunodominant epitopes identified in that study (VLSEWLPVT 121–129 and ILDAHSLYL 165–173) were commercially produced as pentamers and were evaluated for binding in samples from RVFV-vaccinated donors. Notably, the peptides that contained these epitopes were not identified in our ICS or ELISPOT screens of the vaccine recipients. Our initial screens for pentamer-positive CD8 T cells using total PBMCs were negative, so CD8 T cells were isolated by negative selection from 1 × 10^8^ total PBMCs to increase the sensitivity of the assay. Only donor RHI5 and donor RHI2 had tetramer frequencies that were detectable above background (Fig. 4a, b). However, frequencies were too low to reliably phenotype these virus-specific cells.

## Discussion

In this study, humoral and cell-mediated immune responses against a formalin-inactivated RVFV vaccine were measured in humans. This vaccine has been used in laboratory workers since the late 1960s, with anecdotal evidence of protection since no symptomatic laboratory infections were observed after its introduction.^21^ Based upon limited data from animal models, very low plaque reduction neutralization titers of antibodies appear to protect animals from challenge with otherwise lethal doses of RVFV in passive transfer studies.^21^ A published animal study in which a titration of passively transferred antibodies was quantitated prior to challenge reported that a PRNT_80_ titer of 20 was required for complete protection.^22^ A cutoff of 1:40 in a PRNT_80_ assay was chosen as a correlate of protection for use in humans in these early vaccine studies. However, only limited data support this assumption, and the true mechanistic correlates of immune protection against RVFV in humans or animals are not known. Additionally, laboratory workers whose antibody titers were less than 1:10 reportedly worked safely with virus without signs of clinical disease,^21^ and some animals survived challenge with PRNT_80_ < 10,^22^ suggesting that another aspect of immunity, perhaps T cell-mediated immunity, provided protection in this situation.

Work performed by many different groups has established that the type 1 IFN-mediated innate immune response is critical for protection from RVFV in several animal models,^23–27^ but the specific mechanistic roles of the humoral and cell-mediated responses are less well understood. A series of studies performed in the mouse model revealed that monocytes, CD4 T cells, and CD8 T cells played a role in preventing RVFV-mediated encephalitis in the context of an active innate immune response, and that B cells contributed to viral clearance.^12,13^ These key findings suggested that T cells may be involved in modulating RVFV disease, and are further supported by the discovery that HIV-positive patients infected with RVFV had increased case fatality rates and increased occurrence of CNS manifestations of disease.^14,15^

The role of antibodies in disease protection prior to or shortly following exposure to a pathogen is well-established in infectious disease.^28^ The long-standing dogma holds that antibodies prevent viral disease, but T cells are needed to clear viral infection once the disease is manifest. This is exemplified by varicella zoster virus; when administered up to 72 h post exposure, anti-varicella immune globulin lowers disease risk in immunocompromised persons, but once symptoms appear, a robust T cell response is needed to resolve disease.^29^ However, the concept that passively administered antibodies only work to prevent infection has been challenged in recent years. High-dose monoclonal antibody preparations have demonstrated efficacy in treating Ebola virus disease, reducing mortality rates from historical highs of 90% to ~30%.^30^ In Rift Valley fever, pre-exposure passive antibody transfer is well-established to provide protection,^22,31–33^ and one study performed in mice demonstrated that administration of a neutralizing antibody 1 day post infection, but before clinical disease was manifest, improved survival.^34^ However, there are no published studies that address whether antibody transfer is protective once clinical disease is established, and the utility of antibodies as therapy for RVFV infection has not yet been explored.

Major knowledge gaps exist in the field regarding the mechanistic correlates of immune mediated protection during natural RVFV infection. No rigorous studies have evaluated the development of humoral immunity during the course of natural infection in large numbers of patients. Additionally, the function and specificity of T cells during naturally acquired RVFV human disease must be examined to assess correlation with disease severity and outcome. Independently of their role during natural infection, the function of T cells in providing vaccine-mediated protection should also be evaluated.

It was somewhat surprising that a formalin-inactivated vaccine, which typically are not thought to generate strong T cell responses, did generate readily detectable T cell responses in this cohort of RVFV vaccine recipients. Perhaps the fact that many of the donors had received multiple doses of the vaccine contributed to our ability to detect RVFV-specific T cells in these individuals. Another inactivated vaccine that is given repeatedly to humans is the influenza vaccine. However, in the case of influenza, most individuals are likely to encounter influenza in their environment and thus have their responses boosted by that exposure. This is not the case for RVFV vaccine recipients who wear personal protective equipment that should prevent exposure in most circumstances. In a study of human T cell responses to peptides of influenza antigens,^35^ a similar magnitude of spot forming cells following peptide antigen stimulation in IFN gamma ELISPOT assays was observed in healthy humans as we have observed in some of these RVFV vaccine recipients. This suggests that RVFV vaccine antigens (at least the three structural proteins that are present in the formalin-inactivated preparation) are immunogenic. This bodes well for the RVFV vaccine field especially if a platform is used that can generate robust T cell responses with only one dose.

In this report, we established that long-lived, RVFV-specific T cell responses develop in humans exposed to RVFV antigens and were detectable up to 24 years following antigen exposure. The assays described here provide a panel of peptides that could be used in human populations of diverse genetic backgrounds to investigate the T cell response to RVFV N, Gn, and Gc antigens. Since some combination of these antigens is likely to be included in any RVFV vaccine, these assays have broad applicability to studies of RVFV human vaccines.

## Methods

### Institutional Review Board and human subjects

Institutional Review Board approval was granted by the CDC Human Research Protection Office (Protocol #6559). Written informed consent was obtained from all vaccine recipients prior to inclusion. Vaccine recipients provided verbal report of their RVFV vaccination history and had all been vaccinated secondary to occupational exposure risk. PMBCs from normal health controls were obtained via an established normal health phlebotomy protocol (CDC1652).

### Cells and serum

A mononuclear leukapheresis product was obtained from all vaccine recipients and controls. PMBCs were purified over ficoll gradients using standard procedures and then cryopreserved until use. Serum was also obtained from all vaccine recipients and controls using serum separator tubes.

### ELISA

Maxisorp plates (Nalgene-Nunc, Grand Island, NY, USA) were coated with lysate from RVFV-infected Vero-E6 cells or with lysate from uninfected Vero-E6 cells (negative control) diluted 1:2000 in phosphate-buffered saline (PBS)^36^ and allowed to adsorb overnight at 4 °C. Plates were blocked in 5% fetal bovine serum (FBS) and 5% skim milk in PBS 1 h at 37 °C. Patient serum samples were assayed in duplicate and were serially diluted in blocking buffer and then incubated on blocked plates for 1 h at 37 °C. After three washes in PBS with 0.1% Tween 20 (PBST), plates were incubated for 1 h at 37 °C with anti-human IgG HRP (Jackson ImmunoResearch Inc, West Grove, PA, USA) diluted 1:5000 in blocking solution with 0.1% Tween 20. Following 3 PBST washes, the plates were incubated in TMB substrate (KPL) for 10 min; reactions were stopped with the addition of 1% HCl, and plates were read at 450 nm. Data were analyzed using Excel (Microsoft Corp, Redmond, WA, USA) and Prism (GraphPad Software Inc, La Jolla, CA, USA) software. Raw OD values from the negative control Vero-E6 plate were subtracted from those of the RVFV lysate plate. The endpoint titer was defined as the dilution of the serum that gave a value at least three standard deviations above the average value obtained from the negative control serum.

### FRNT_50_ assay

Serum samples from vaccine recipients and from an unvaccinated control human donor were heat-inactivated at 56 °C for 30 min prior to serial dilution in DMEM supplemented with 10% FBS. An equal volume of DelNSs/DelNSm:ZsG RVFV at concentration of 8000 FFU/mL was mixed with the diluted sera and samples were incubated for 1 h at 37 °C. 100 µL of these mixtures were then inoculated onto 96-well plates of Vero-E6 cells and incubated for 1 h at 37 °C. After adsorption, the inoculum was removed, and the cells were overlaid with 1.5% carboxymelthylcellulose (CMC) in MEM10. Following an overnight incubation at 37 °C, plates were washed three times with PBS prior to fixation with 10% formalin. Cells were then permeabilized with 0.1% TritonX100 in PBS for 10 min at room temperature (RT), washed again, and blocked in 5% non-fat milk diluted in PBST for 20 min at RT. Cells were then incubated with a rabbit polyclonal anti-RVFV N antibody (custom product, Genscript, USA) diluted 1:500 in 5% milk-PBST for 2 h at RT. After 2 PBS washes, cells were incubated for 1 h at RT with an HRP-conjugated anti-rabbit IgG antibody (Invitrogen, Thermo Fisher Scientific) diluted 1:500 in 5% milk-PBST. Following 2 PBS washes, the cells were incubated at RT with KPL HistoMark TrueBlue TMB substrate (SeraCare) until foci were clearly visualized. Plates were washed twice with MilliQ water and allowed to dry fully before data were acquired on an Immunospot Analyzer (C.T.L., Cleveland, OH). Foci were counted in control wells and compared to foci numbers in experimental wells. The dilution of serum at which 50% of foci are neutralized is reported as the FRNT_50_.

### Intracellular cytokine staining peptide mapping assays

Peptides were made to >70% purity and confirmed by HPLC (Genscript, USA). Lyophilized peptides were suspended in DMSO to 10 mg/mL stocks and stored at −20 °C. Cryopreserved PBMCs were thawed and washed in RPMI medium with 10% FBS; 1 × 10^6^ cells were incubated with each peptide pool (2 µg of each peptide/mL) in the presence of CD49d and CD28 (1 µg of each/mL) and Brefeldin A (10 µg/mL) for 6 h at 37 °C in RPMI medium with 10% FBS. Negative control PBMCs from unvaccinated normal healthy controls were included in all assays. Cells were washed in PBS, stained with Near-IR Live/Dead stain (diluted 1:500 in PBS) for 10 min, and washed in flow buffer (PBS with 2% FBS). Following addition of Fc block, cells were stained with a surface mix of CD3 PCPCy5.5 (Cat #340949, SK7), CD4 APC (Cat#555349, RPA-T4), CD8 BV510 (Cat#563919, SK-1), and CD45RA PECy7 (Cat#560675, HI100) for 20 min followed by 2 flow buffer washes. All antibodies used in flow cytometric assays were used at the dilution recommended by the manufacturer. Cells were then treated with Cytofix/Cytoperm (BD) for 20 min at RT followed by 2 washes in Perm/Wash and 30 min incubation with the intracellular antibody mix IFN-γ FITC (Cat#340449, 25723.11), TNF-α PE (Cat#559321, MAb11), and IL-2 BV421(Cat#562914, 5344.111) followed by 2 Perm/Wash changes. Data were then acquired on a Stratedigm S1000EXi and analyzed using Flow Jo; gates were set based on negative control samples. An example of the gating strategy used in all flow cytometric assays is shown in the supplementary material. Data were exported to Excel and peptide pools were considered positive when cytokine expression frequency was greater than zero after subtracting the signal from the negative controls for any of the intracellular staining markers.

### ELISPOT peptide assays

Cryopreserved PBMCs were thawed and washed in RPMI medium with 10% FBS, and 3 × 10^5^ cells were incubated with each peptide (individually or in pools; 2 µg of each peptide/mL) in a precoated human IFN-γ ELISPOT plate (Mabtech) overnight in RPMI medium with 10% FBS. Negative control PBMCs from unvaccinated normal healthy controls were run in all assays. Following overnight incubation, plates were processed according to manufacturer’s instructions. Spots were counted using an Immunospot Analyzer. Data were exported to Excel, and in initial assays, peptide pools were considered positive when spot count was greater than zero after subtracting signal from the negative controls. For single peptide assays, the number of spots was recorded per 1 × 10^5^ cells, and data were entered into Prism to generate plots.

### Evaluation of T cell activation and proliferation following stimulation with down-selected peptides for each viral antigen

Cryopreserved PBMCs were thawed and washed in RPMI medium with 10% FBS, and 4 × 10^7^ cells from each vaccine recipient and a negative control donor were stained with CellTrace Violet (Thermo Fisher) according to manufacturer’s instructions. After washing, 4 × 10^6^ cells were suspended in RPMI medium with 10% human AB serum and incubated with pools of down-selected peptides from each antigen (5 µg each peptide/mL), or with IL-7/IL-15 (50 ng/mL) or left unstimulated. After a 3 d incubation at 37 °C, cells were washed in PBS and stained with Near-IR Live/Dead as described above. Following the addition of Fc block, cells were incubated for 30 min at RT with the following surface stains: CD3 PCPCy5.5 (SK7), CD4 BV786 (Cat#563881, SK2), CD8 APC (Cat#301014, RPA-T8), CD14 APCCy7 (Cat#561709, MphiP9), CD19 APCCy7 (Cat#557791, SJ25C1), and CD69 PE (Cat#555531, FN50). After 2 flow buffer washes, cells were treated with Cytofix/Cytoperm for 20 min at RT, washed twice in Perm/Wash, and incubated with Ki-67 488 (Cat#561165, B56) for 45 min followed by two changes of Perm/Wash. Data were then acquired on a Stratedigm S1000EXi and analyzed using Flow Jo; gates were set based on negative control samples.

### Intracellular cytokine assays to assess functionality using down-selected peptides for each antigen

Cryopreserved PBMCs were thawed and washed in RPMI medium with 10% FBS, and 2 × 10^7^ cells were incubated with a pool of down-selected peptides from each antigen (2 µg each peptide/mL) in the presence of CD49d and CD28 (1 µg/mL each), CD107a PE (Cat#560948, H4A3), and Brefeldin A (10 µg/mL) for 6 h at 37 °C in RPMI medium with 10% FBS. Cells were also incubated with DMSO vehicle as a negative control and SEB (1 µg/mL) as a positive control. Negative control PBMC from three unvaccinated normal healthy individuals were included in the assays. Cells were washed in PBS, stained with Near-IR Live/Dead (diluted 1:500 in PBS) for 10 min, and washed in flow buffer. Following Fc block, cells were incubated for 20 min with CD3 PCP(Cat#552851, SP34-2), CD4 BV786 (SK2), CD8 BV711 (Cat#563676, RPA-T8), CD14 APCCy7 (MphiP9), and CD19 APCCy7 (SJ25C1) before 2 flow buffer washes. Cells were then treated with Cytofix/Cytoperm for 20 min at RT followed by 2 washes in Perm/Wash and incubation with the intracellular antibodies IFN-γ FITC (25723.11), CD154 BV605 (Cat#310826, 24–31), TNF-α APC (Cat#502912, MAb11), and IL-2 BV421 (5344.111) for 45 min followed by two washes in Perm/Wash. Data were then acquired on a Stratedigm S1000EXi and analyzed using Flow Jo; gates were set based on the negative control samples.

### Pentamer staining in HLA-A2 donors

HLA-A2 pentamers (Proimmune) were generated for the peptides VLSEWLPVT 121–129 and ILDAHSLYL 165–173 derived from the N protein. Both APC- and PE-labeled pentamers were used. The HLA-A2 Flu tetramer corresponding to peptide sequence GILGFVFTL was kindly provided by Rama Akondy. Cryopreserved PBMCs from vaccine recipients and from a negative control HLA-A2 + donor were thawed and washed in RPMI10, and CD8 T cells were purified using negative selection from 1 × 10^8^ cells according to manufacturer’s instructions (Miltenyl). Purified CD8 T cells (5 × 10^6^) or total PBMCs (5 × 10^6^) were washed in PBS, stained with Near-IR Live/Dead as described above, and washed in flow buffer prior to incubation with pentamers (50 µL APC-labeled and PE-labeled) for 30 min at RT. Cells were then washed with flow buffer and Fc block was added prior to 45 min RT incubation with surface stain: CD8 BV711 (RPA-T8), CD45RA PECy7 (HI100), CCR7 BV510 (Cat#353232, G043H7), CD14 APCCy7 (MphiP9), CD19 APCCy7 (SJ25C1), PD-1 BV421 (Cat#329920, EH12.2H7), CD28 BV605 (Cat#562976, CD28.2), and CD27 FITC (Cat#302806, O323). Following two additional washes, data were acquired on a Stratedigm S1000EXi and analyzed using Flow Jo; gates were set based on negative control samples.

### Reporting summary

Further information on experimental design is available in the Nature Research Reporting Summary linked to this article.

## Supplementary information

Supplementary Figure

Reporting Summary

## Data Availability

All data generated or analyzed during this study are included in this published article.
