# Supplementary material for: Rift Valley fever virus vaccination induces long-lived, antigen-specific human T cell responses
Source: NPJ Vaccines. 2020 Feb 28;5:17. doi: 10.1038/s41541-020-0166-9 (PMC7048758; doi:10.1038/s41541-020-0166-9)
Supplement: Supplementary file 1 — Supplementary Figure [file 41541_2020_166_MOESM1_ESM.pdf]

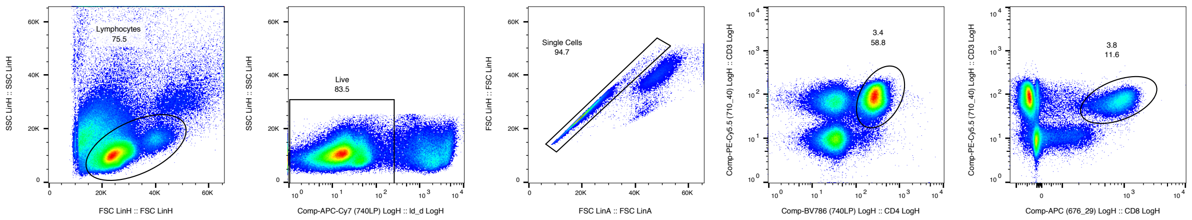

Supplementary Figure: The gating strategy depicted was used in all flow cytometric analyses. Lymphocytes were gated followed by Live gating, singlet gating then specific gating on either CD3+CD4+ cells or CD3+CD8+ cells. All downstream analyses are shown in the manuscript figures.
